# Draft genome sequences of *Thermus thermophilus* strains isolated from Japanese hot springs

**DOI:** 10.1128/mra.00559-25

**Published:** 2025-06-20

**Authors:** Yu Sato, Masanori Hashino, Shintaro Maeno

**Affiliations:** 1 Research Center for Thermotolerant Microbial Resources, Yamaguchi University 13150, Yamaguchi, Japan; 2 Laboratory of Bacterial Genomics, Pathogen Genomics Center, National Institute of Infectious Diseases, Tokyo, Japan; California State University San Marcos, San Marcos, California, USA

**Keywords:** draft genome, *Thermus thermophilus*, isolates, hot spring

## Abstract

*Thermus thermophilus* is one of the most studied thermophilic bacteria. In this study, we report the draft genomes of four strains newly isolated from Japanese hot springs. The phylogenetic analysis of *T. thermophilus* strains isolated from diverse environments suggests a link between geographical distribution and genomic diversity.

## ANNOUNCEMENT

Thermophilic bacteria belonging to the genus *Thermus* are among the most studied thermophiles, which can proliferate with temperatures ranging from 37 to 85°C (1). In particular, *Thermus thermophilus* has been found from diverse environments, including hot springs (2–8), a deep-sea hydrothermal vent (9), a geothermal field located in an alpine mountain (10), and even artificial thermal habitats, such as cheese (11, 12), power plants (13), and compost (14). These wide-ranging habitats make *T. thermophilus* an excellent model for studying environmental adaptation, particularly thermal adaptation (15–19). Furthermore, their biomolecules have been structurally analyzed (20, 21) and have potential for the development of new tools in biotechnology (22–24). Therefore, increasing the number of *T. thermophilus* strains is important for both basic physiological and ecological research and industrial applications.

In this study, we isolated four *T. thermophilus* strains, that is, KIK1 and KIK4 isolated from Kaike hot spring (35.45°N, 133.36°E) and REK1 and REK4 from Obama hot spring (32.72°N, 130.20°E) in Japan and provided their draft genome sequences. We directly took 200 mL of water sample from each hot spring in sterilized bottles. One milliliter of hot spring water was streaked onto R2A medium (DSMZ Medium 830) supplemented with 1% (v/v) SL-6 trace metal solution and incubated at 60°C for 48 h. The same procedure was performed three times to ensure isolation of yellow colonies. Each isolate was cultured in the same medium at 65°C overnight, followed by DNA extraction using the Wizard Genomic DNA Purification Kit (Promega, Madison, WI, USA). Short-read sequencing libraries were prepared using the QIAseq FX DNA Library UDI-B Kit (96) (Qiagen, Hilden, Germany). These DNA libraries were sequenced on the NextSeq 2000 platform (Illumina, San Diego, CA, USA) to generate paired-end sequences (151 bp × 2). The Illumina reads underwent trimming and quality control using Platanus_trim v1.0.7 and assembled using Platanus_b v1.3.2 (25). Contigs shorter than 200 bp were eliminated. These assembled sequences were annotated using the DDBJ Fast Annotation and Submission Tool (DFAST) v1.3.1 (26). The completeness and contamination rates of the genomic data were assessed using CheckM v1.2.2 (27) in the DFAST program. Taxonomic assignment was also performed using the taxonomy check function of DFAST. All isolates showed average nucleotide identity values > 95% with reference genomes of *T. thermophilus*. Default parameters were used for all software.

**Fig 1:**
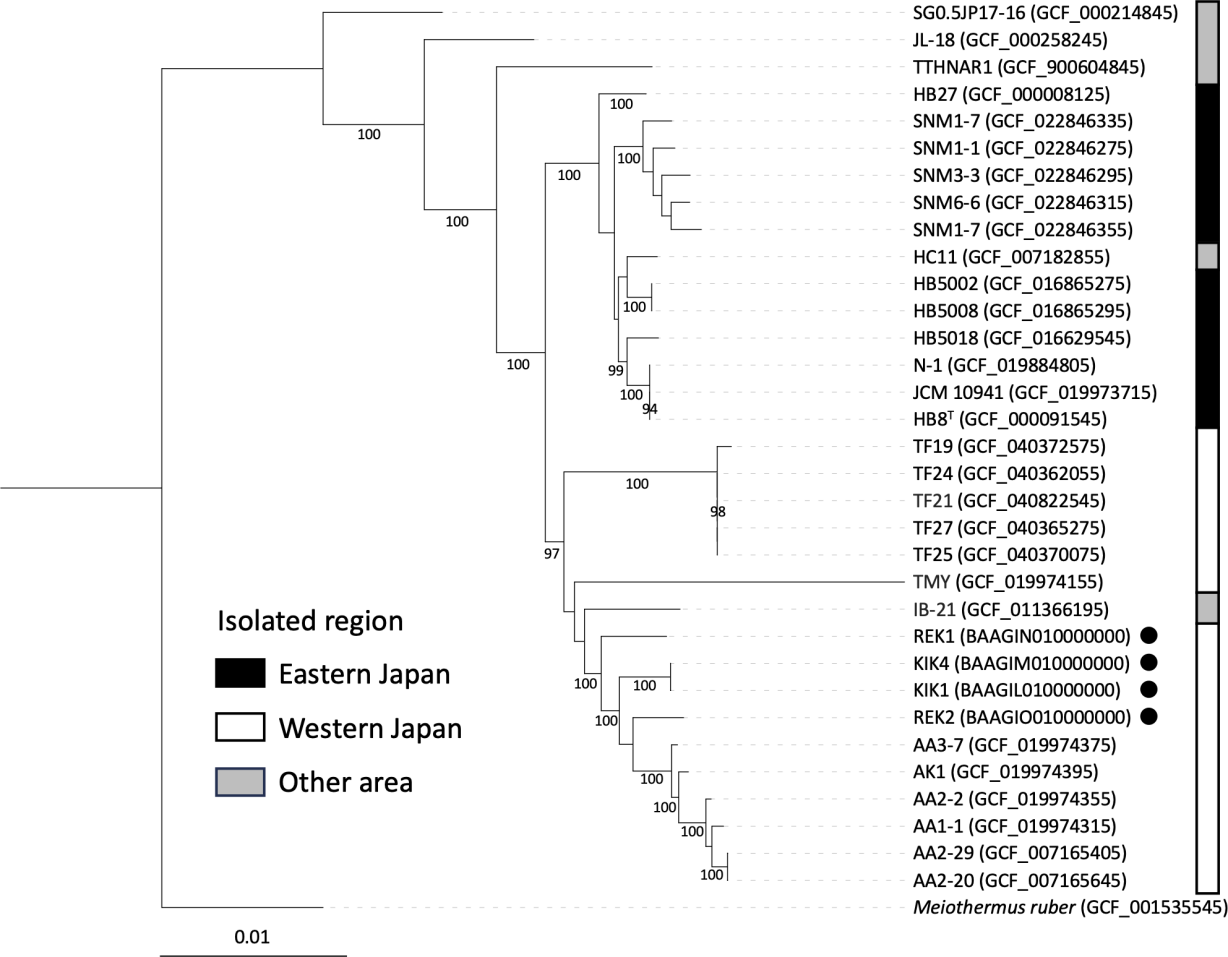
Maximum likelihood tree of 81 core genes among strains of *T. thermophilus*. Labels for nodes with less than 80% bootstrap support were removed. The isolates in this study are highlighted with filled circles. The scale bar represents nucleotide substitutions per site. According to the geographical location of isolation, the strains were classified into the following three groups: black bar, east side of the Itoigawa-Shizuoka Tectonic Line (ISTL); white bar, west side of the ISTL; and gray bar, other areas. *Meiothermus ruber* RL^T^ was used as the outgroup. Bar, 0.01 substitutions.

The details of the draft genomes for *T. thermophilus* strains in this study are summarized in Table 1. The four genomes showed a completeness value ranging from 95.1 to 96.6, with contamination ranging from 0.6 to 2.9. A phylogenetic analysis was performed based on the concatenation of 81 core genes from each genome using UBCG2 (28). The phylogenetic analysis indicates *T. thermophilus* strains found in Japan were divided into two clades: the west and east sides of the Itoigawa-Shizuoka Tectonic Line (Fig. 1). Additionally, the four strains (KIK1, KIK4, REK1, and REK2) isolated in this study were closely related to the strains from Arima hot springs in the westside. This study reveals the relationship between the geographical distribution and the genomic diversity of *T. thermophilus*.

**TABLE 1:** Information on the draft genome sequences of *T. thermophilus* isolates from this study and the type strain HB8

Strain	KIK1	KIK4	REK1	REK2	HB8^T^
TYGS dDDH (d4%) (with the type strain HB8)	78.4	78.2	76.9	77.6	NA^*a*^
Average nucleotide identity (with the type strain HB8)	97.5	97.6	97.6	97.4	NA^*a*^
Assembly length (bp)	2,280,537	2,280,474	2,497,999	2,461,630	2,116,056
No. of contigs	277	240	184	161	3
G + C (%)	69.1	69.1	68.8	68.9	69.5
Contigs N50 (bp)	35,737	51,860	73,412	73,281	1,849,742
CDS	2,320	2,354	2,606	2,566	2,238
rRNA	6	4	3	2	6
tRNA	52	51	51	52	54
CRISPR	9	5	15	5	10
Coding ratio (%)	89.1	90.8	90.9	91.6	94.6
CheckM completeness (%)	95.8	95.8	95.1	96.6	97.6
CheckM contamination (%)	0.6	0.6	2.4	2.9	0.1
Total no. of reads	12,414,390	6,506,566	11,115,028	2,535,252	NA^*a*^
SRA accession no.	DRX620850	DRX620851	DRX620852	DRX620853	NA^*a*^
GenBank accession no.	BAAGIL010000000	BAAGIM010000000	BAAGIN010000000	BAAGIO010000000	GCA_000091545.1

^*a*^ Not analyzed in this study.

## Data Availability

The draft genome sequences of four strains are deposited at DDBJ/EMBL/GenBank under accession numbers BAAGIL010000000, BAAGIM010000000, BAAGIN010000000, and BAAGIO010000000. Trimming data are available in the Sequence Read Archive (SRA) under accession numbers DRX620850, DRX620851 , DRX620852, and DRX620853.
